# Ag85B with c-di-AMP as mucosal adjuvant showed immunotherapeutic effects on persistent *Mycobacterium tuberculosis* infection in mice

**DOI:** 10.1590/1414-431X2024e13409

**Published:** 2024-07-01

**Authors:** Xuan Liang, Ruonan Cui, Xue Li, Huanhuan Ning, Jian Kang, Yanzhi Lu, Shan Zhou, Xinying Huang, Yujun Peng, Jingyao Zhang, Shiyun Li, Yanling Ma, Yinlan Bai

**Affiliations:** 1 College of Life Sciences, Northwest University, Xi'an, China; 2 Department of Microbiology and Pathogen Biology, Air Force Medical University, Xi'an, China; 3 Military Medical Innovation Center, Air Force Medical University, Xi'an, China; 4 Department of Clinical Laboratory, Xijing Hospital, Air Force Medical University, Xi'an, China; 5 Student Brigade, Basic Medical School, Air Force Medical University, Xi'an, China

**Keywords:** Mycobacterium tuberculosis, Ag85B, C-di-AMP, Adjuvant, Immunotherapy

## Abstract

Tuberculosis (TB), caused by *Mycobacterium tuberculosis*, remains the leading cause of mortality by a single infectious agent in the world. *M. tuberculosis* infection could also result in clinical chronic infection, known as latent TB infection (LTBI). Compared to the current limited treatment, several subunit vaccines showed immunotherapeutic effects and were included in clinical trials. In this study, a subunit vaccine of Ag85B with a novel mucosal adjuvant c-di-AMP (Ag85B:c-di-AMP) was delivered intranasally to a persistent *M. tuberculosis* H37Ra infection mouse model, which also presented the asymptomatic characteristics of LTBI. Compared with Ag85B immunization, Ag85B:c-di-AMP vaccination induced stronger humoral immune responses, significantly higher CD4^+^ T cells recruitment, enhanced Th1/Th2/Th17 profile response in the lung, decreased pathological lesions of the lung, and reduced *M. tuberculosis* load in mice. Taken together, Ag85B:c-di-AMP mucosal route immunization provided an immunotherapeutic effect on persistent *M. tuberculosis* H37Ra infection, and c-di-AMP, as a promising potential mucosal adjuvant, could be further used in therapeutic or prophylactic vaccine strategies for persistent *M. tuberculosis* infection as well as LTBI.

## Introduction

*Mycobacterium tuberculosis* is the causative agent of tuberculosis (TB), which is the second leading infectious cause of death globally after COVID-19. In 2021, over 10.0 million people developed the active form of TB and 1.6 million people died of TB (1,2). As an intracellular parasite, *M. tuberculosis* establishes a long-lasting infection in the host after infection, resulting in clinical chronic infection or latent TB infection (LTBI), which can persist for decades, or in symptomatic ‘active tuberculosis', and result in further transmission (3). The emergence of resistance to first-line antibiotic poses a threat to successful treatment, and more data are needed on efficacy and safety of chemotherapy of LTBI treatments (4). Thus, novel therapeutic options are required, particularly for drug-resistant tuberculosis.

The new subunit vaccines account for two-thirds of the current clinical vaccines, including ID93+GLA-SE, H56:IC31, and M72/AS01E, which are comprised of *M. tuberculosis* proteins with immunodominant human T cell epitopes and are formulated with different mechanism adjuvants (5). New immunotherapies with immunodominant antigen and new adjuvants should be investigated in the preclinical research stage for future clinical application.

Ag85B, mainly secreted by *M. tuberculosis* in early growth, possesses mycolyl-transferase and fibronectin binding activity (6). Ag85B contains multiple epitopes that could elicit humoral as well as Th1 or Th17 cellular immune responses, which provides a long-term protective response against *M. tuberculosis* infection (7). Until now, six TB vaccine candidates are composed of Ag85A or Ag85B, such as H56:IC31 (Ag85B, ESAT-6, Rv2660) (8). Ag85B-specific CD4^+^ T cell responses induced by H56:IC31 were highly persistent and sustained even up to 292 days (9). Thus, more efforts should be made to improve efficiency and safety of Ag85B-based vaccine of TB immunotherapy.

Cyclic dimeric adenosine monophosphate (c-di-AMP) is a second messenger derived from bacteria involved in several physiological processes (10,11). Moreover, c-di-AMP could activate innate immune responses, including type I interferon signaling, autophagy, and inflammation (12,13). Our previous work has demonstrated that c-di-AMP, as a mucosal adjuvant, could enhance the immunogenicity of ESAT-6, a vaccine candidate antigen of *M. tuberculosis*, which provided protection against *M. tuberculosis* challenge (14). As mucosal adjuvant, c-di-AMP enhanced the immunogenicity of pathogen-specific antigens and induced stronger Th1/Th2/Th17 immune responses (15). Further, subcutaneous immunization of guinea pigs with recombinant BCG with c-di-AMP as an endogenous adjuvant also provided immune protection against *M. tuberculosis* (16), similar to what we found in mice immunized with recombinant BCG (17). Thus, c-di-AMP can be a safe and effective adjuvant of subunit vaccines for mucosal delivery.

In this study, we investigated the immune responses induced by Ag85B with c-di-AMP as a mucosal adjuvant (Ag85B:c-di-AMP) and evaluated its immunotherapy effect on persistent *M. tuberculosis* H37Ra infection in a mouse model that contained stable bacteria and immune responses to *M. tuberculosis* until the mice were old but without any symptoms, as a phenotype similar to LTBI.

## Material and Methods

### Ethics statement

Animal studies were conducted under the recommendations from the Guide for the Care and Use of Laboratory Animals of Department of Lab Animal Science, Air Force Medical University, China) (TDL 20190213). The serum of TB patients was collected at the Xi'an Chest Hospital of Shannxi province after the patients signed an informed consent. Sera of mice and guinea pigs infected with *M. tuberculosis* H37Rv were prepared and preserved by our research group (18,19). The studies were reviewed and approved by Institutional Ethics committee of Air Force Medical University (KY 20194021).

### Animals and bacterial strains

Female BALB/c mice (6 to 8 weeks old) were purchased from the Animal Center of Air Force Medical University (China). *M. tuberculosis* H37Ra strain was obtained from the National Institutes for Food and Drug Control in China. *M. tuberculosis* H37Ra was grown in Middlebrook 7H9 medium (BD, USA) supplemented with 0.5% glycerol, 10% oleic acid-albumin-dextrose-catalase (OADC) (BD), and 0.05% Tween-80 (Sigma, USA) at 37°C. The log-phase bacteria were harvested and colony-forming units (CFUs) were counted using Middlebrook 7H10 plates supplemented with OADC.

### Purification of Ag85B protein

The prokaryotic expression plasmid pProEXHTb-Ag85B was constructed and preserved by our research group. Recombinant *E. coli* DH5α strain carrying pProEXHTb-Ag85B was induced with 0.5 mM isopropyl β-D-1-thiogalactopyranoside (IPTG) for 4 h at 37°C. Then, Ag85B protein was purified by using Ni^2+^ affinity chromatography as reported previously (20). Finally, the protein was dialyzed with gradient concentration urea and PBS, quantified by BCA assay, and stored at -30°C (Supplementary Figure S1).

### Mouse infection and immunization

**Figure 1:**
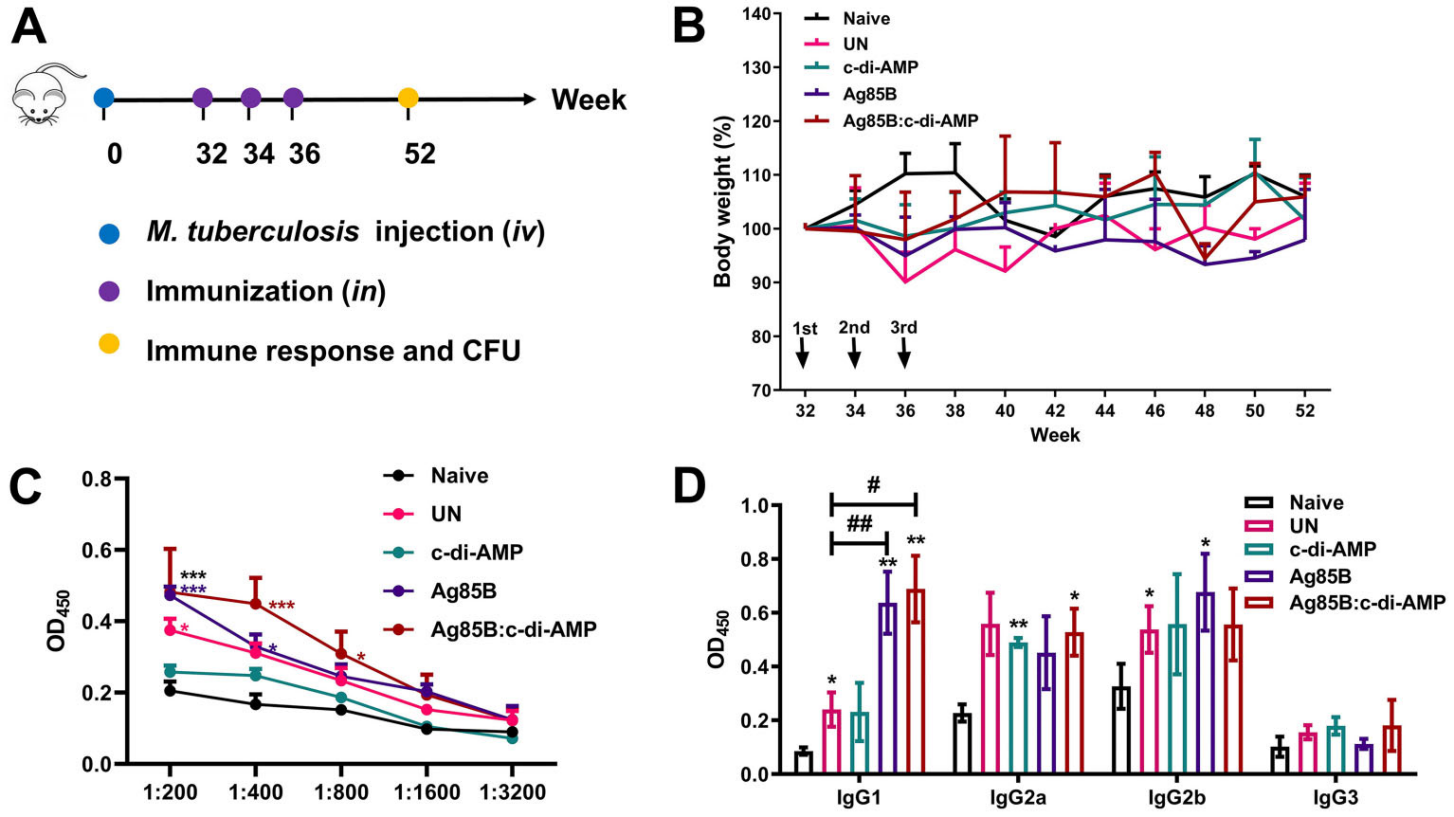
The subunit vaccine Ag85B:c-di-AMP induced systemic IgG in *M. tuberculosis*-infected mice. **A**, *M. tuberculosis* infection (*iv*) and intranasal vaccination (*in*) schedule. Female BALB/c mice infected with 5×10^4^ *M. tuberculosis* H37Ra through *iv* route. At 32-week post infection, mice were immunized with c-di-AMP, Ag85B, or Ag85B:c-di-AMP three times by *in* route at a 2-week interval. Naive mice and *M. tuberculosis*-infected mice without immunization (UN) received PBS. At 52 weeks, immune responses and colony forming units (CFUs) were determined (n=3). **B**, Mice were weighed at 2-week intervals. Data are reported as percentage of initial weight at 32 weeks. Vaccination is indicated with black arrows (n=3). **C**, Ag85B-specific IgG titers in serum at 52 weeks detected by ELISA at indicated dilutions (1:200 to 1:3 200) (n=3). **D**, Ag85B-specific IgG subclasses in serum (1:200) of mice at 52 weeks assayed by ELISA (n=3). Data are reported as means ± SE. *P<0.05, **P<0.01, ***P<0.001 compared to Naive (control); ^#^P<0.05, ^##^P<0.01 compared to UN (infected unimmunized); Student's *t*-test.

Mice were infected with 5×10^4^ CFUs of *M. tuberculosis* H37Ra in 0.2 mL PBS by intravenous (*iv*) injection (*iv*) (21). The control mice were inoculated with the same volume of PBS (Naive). At 32 weeks, mice were randomly divided into 4 groups to receive intranasal (*in*) immunization as follows: *M. tuberculosis-*infected mice without immunization (UN) receiving 50 µL saline, Ag85B group receiving 30 µg Ag85B, c-di-AMP group receiving 5 µg c-di-AMP, and Ag85B:c-di-AMP group receiving 30 µg protein and 5 µg c-di-AMP. Intranasal application was performed twice at 2-week intervals. Mice were observed for 52 weeks (Figure 1A). The body weights of mice were monitored at 2-week intervals throughout the experiment. The results of body weight are reported as the percentage of initial weight at first infection/intranasal vaccination.

### Detection of antibody levels by ELISA

Serum was diluted as indicated and added in Ag85B-coated wells to incubate for 1 h at room temperature. After washing, 100 µL/well of HRP-conjugated goat anti-mouse IgG, IgG1, IgG2a, IgG2b, IgG3 (Affinity Biosciences, USA) (1:5000) were used as detection antibodies and incubated for 45 min at 37°C. Subsequently, 3,3',5,5'-tetramethylbenzidine (TMB) was added and the reaction was stopped with 2 M H_2_SO_4_. Absorbance was recorded at 450 nm using a microplate reader (BioTek, USA).

### Flow cytometry

Splenocytes were stimulated with Ag85B proteins (5 µg/mL) for 24 h at 37°C with 5% CO_2_. For cytokine analyses, protein transport inhibitor of Brefeldin A (BioLegend, USA) was added and incubated for another 12 h. Cells were collected and resuspended in 100 µL Staining Buffer (BioLegend). Anti-CD32/16 monoclonal antibodies (mAbs) (BioLegend) were added to block Fc receptors. Surface markers were stained with CD4 and CD8 mAbs. Subsequently, cells were fixed and permeabilized using the Cytofix/Cytoperm Fixation/Permeabilization Solution (BD). Then, the intracellular cytokines were labeled with interferon (IFN)-γ, interleukin (IL)-2, and IL-10 mAbs and incubated for 30 min at 4°C. Cells were washed using Perm/Wash buffer and resuspended in 500 µL Staining Buffer for flow cytometry. Data were analyzed using FlowJo software (TreeStar, USA).

### Real-time quantitative PCR

Total RNA from lung tissue of mice was extracted using the RNA kit (OMEGA, USA) following the manufacturer's protocol. cDNA was synthesized by using HiScript Reverse Transcriptase Kit (Vazyme, China). A real-time quantitative PCR assay was performed using Taq Pro Universal SYBR qPCR Master Mix (Vazyme). The relative expressions of mRNA were calculated according to 2^-ΔΔCT^ with GAPDH as the reference gene. Primer sequences used in this study were based on previous work (14).

### Cytokine measurement by ELISA

Splenocytes were stimulated with Ag85B (5 µg/mL) for 72 h at 37°C with 5% CO_2_. Supernatants were harvested and stored at -20°C. Cytokine production of IFN-γ, IL-2, and IL-10 were assayed using ELISA kits (Thermo Fisher Scientific, USA) according to the manufacturer's instructions. The concentrations of cytokines were determined using curves from standard substances.

### Histopathology and immunohistochemistry

The lower lobe of the left lung was removed and fixed in 4% paraformaldehyde. The tissues were then dehydrated, embedded in paraffin, cut into 5-µm sections and stained with HE. The pathological changes of lung sections such as peri-bronchiolitis, perivasculitis, alveolitis, and granuloma were quantified and scored as 0, 1, 2, 3, 4, or 5 for absent, minimal, slight, moderate, marked, or strong according to Dormans' research, and the final score was the sum of the sub-scores (22). To analyze T cell aggregation, mAbs of anti-CD4 (1:100, Cell Signaling Technology, USA) and anti-CD8 (1:400, Cell Signaling Technology, USA) were stained using immunohistochemistry (IHC). IHC assay was performed by Chengdu Lilai Biotechnology Company (China). The slices were observed under a microscope at 40×10 magnification (Olympus, Japan). The percentages of CD4^+^ and CD8^+^ T cells in the lung were quantified using the ImageJ software (NIH, USA).

### CFU counting

Mice were sacrificed, and spleens and lungs were removed aseptically and homogenized through a 40-µm cell strainer. Serial dilutions were smeared on 7H10 plates supplemented with 10% OADC. Agar plates were incubated at 37°C for 3 weeks. Colonies were counted, and bacteria burdens are reported as log_10_ CFU.

### Statistical analysis

Statistical analysis was performed using GraphPad Prism 5.0 software (USA). Each experiment was performed at least in triplicate. All data except bacteria burdens are reported as means±SE. P-values were determined by Student's *t*-test. Differences with P-values less than 0.05 were considered statistically significant.

## Results

### IgG levels of Ag85B increased in different *M. tuberculosis*-infected hosts

**Figure 2:**
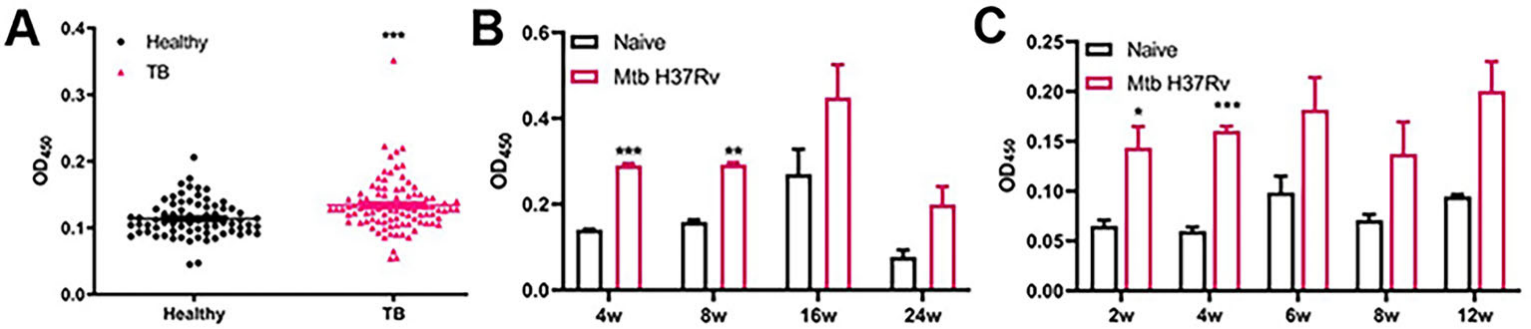
Ag85B-specific IgG in TB patients and *M. tuberculosis*-infected animals. **A**, Ag85B-specific IgG in sera (1:50) of healthy individuals (n=72) and TB patients (n=96). **B** and **C**, Ag85B-specific IgG (1:200) in sera of *M. tuberculosis*-infected mice (**B**) and guinea pigs (**C**) (n=3) post infection at indicated time points (n=3). *P<0.05, **P<0.01, ***P<0.001 compared to healthy (**A**) or Naive (**B** and **C**) animals; Student's *t*-test.

In this study, Ag85B was purified using nickel affinity chromatography (18). We found that the IgG levels against Ag85B were significantly higher in TB patients than non-infected subjects (P<0.001) (Figure 2A). These data were in line with a previous study that reported higher Ag85B-specific antibody levels in *M. tuberculosis* infection individuals than that of non-infected (23). Increased IgG levels against Ag85B were observed after *M. tuberculosis* infection in mice within 8 weeks and in guinea pigs within 4 weeks (Figure 2B and C). These results showed that as an early secretory protein and marker of bacterial reproduction, Ag85B from *M. tuberculosis* continuously stimulated the immune system during infection. Therefore, we speculated that a vaccine designed based on Ag85B could induce immune response to block the bacterial role in the pathogenic process during *M. tuberculosis* infection.

### Ag85B:c-di-AMP triggered humoral immune responses in *M. tuberculosis*-infected mice

Intravenous *M. tuberculosis* infection caused about 20% weight loss after 30 weeks of infection compared to normal mice, and then weight was maintained with no more than 10% variation until the end of the experiment (Supplementary Figure S2). Ag85B and c-di-AMP did not cause significant weight changes in *M. tuberculosis*-infected mice compared with the UN group and caused a tendency for weight loss in infected mice (Figure 1B). These data suggested that intranasal immunization of Ag85B or c-di-AMP was safe for elder mice infected with *M. tuberculosis*.

Anti-Ag85B antibody level was elevated in *M. tuberculosis*-infected mice. Ag85B and Ag85B:c-di-AMP immunization induced higher IgG and IgG1, and Ag85B:c-di-AMP induced higher IgG levels than Ag85B immunization (Figure 1C and D). These data demonstrated that Ag85B intranasal vaccination could induce stronger specific antibodies in *M. tuberculosis*-infected mice and confirmed that c-di-AMP as an adjuvant could improve the immunogenicity of Ag85B, which means reduced amount of antigen used.

### Ag85B:c-di-AMP immunization regulated cellular immune responses in spleen

**Figure 3:**
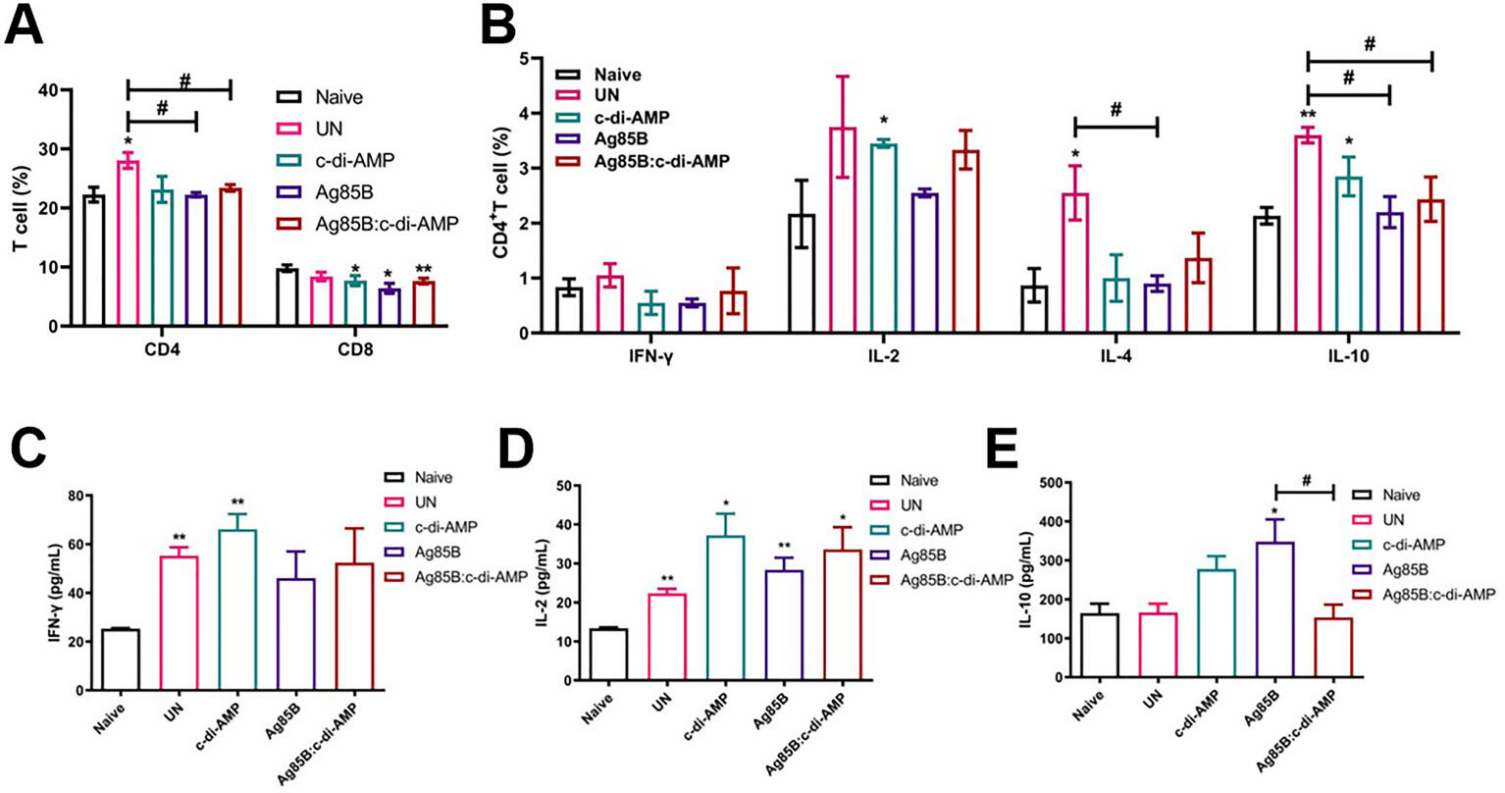
Cellular immune responses in spleen of *M. tuberculosis*-infected mice. **A**, Percentages of CD4^+^ and CD8^+^ T cells in splenocytes detected by flow cytometry (n=3). **B**, After splenocytes were stimulated with Ag85B (5 μg/mL) for 24 h, the proportions of CD4^+^ T cells producing interferon (IFN)-γ, interleukin (IL)-2, IL-4, and IL-10 were analyzed by flow cytometry (n=3). **C**-**E**, At 52 weeks, splenocytes were stimulated with Ag85B (5 μg/mL) for 72 h *in vitro*, and supernatants were collected for IFN-γ, IL-2, and IL-10 cytokine measurement by ELISA. Data are reported as means±SE. *P<0.05, **P<0.01 compared to Naive (n=3); ^#^P<0.05 as indicated; Student's *t*-test.

Here, *M. tuberculosis* infection also increased the proportion of CD4^+^ T cells but not CD8^+^ T cells compared to the UN mice in splenocytes (P<0.05). Both Ag85B and Ag85B:c-di-AMP administration reduced the proportion of CD4^+^ T cells compared to the UN group in splenocytes (P<0.05), and all immunization groups showed decreased proportions of CD8^+^ T cells compared to Naive mice (Figure 3A). *M. tuberculosis* infection led to a significant increase of IL-4 (P<0.05) and IL-10 (P<0.01), and a tendency for higher proportion of Th1 cytokines secreted by CD4^+^ T cells compared to the Naive mice. Noticeably, all immunization groups (Ag85B and c-di-AMP alone or combined) showed a tendency for a lower proportion of both Th1 and Th2 cytokines profile secreted by CD4^+^ T cells in splenocytes compared with the UN group (Figure 3B). Further, it was shown that *M. tuberculosis* infection increased Th1 cytokine secretion but not Th2 cytokine secretion compared with the Naive group. Ag85B and c-di-AMP immunization alone increased Th1 and Th2 cytokines profile compared with the Naive group. However, Ag85B:c-di-AMP immunization did not modulate IFN-γ secretion but inhibited IL-4 secretion by CD4^+^ T cells in splenocytes (Figure 3C-E).

### Ag85B:c-di-AMP immunization induced CD4^+^ T cells recruitment in lung

**Figure 4:**
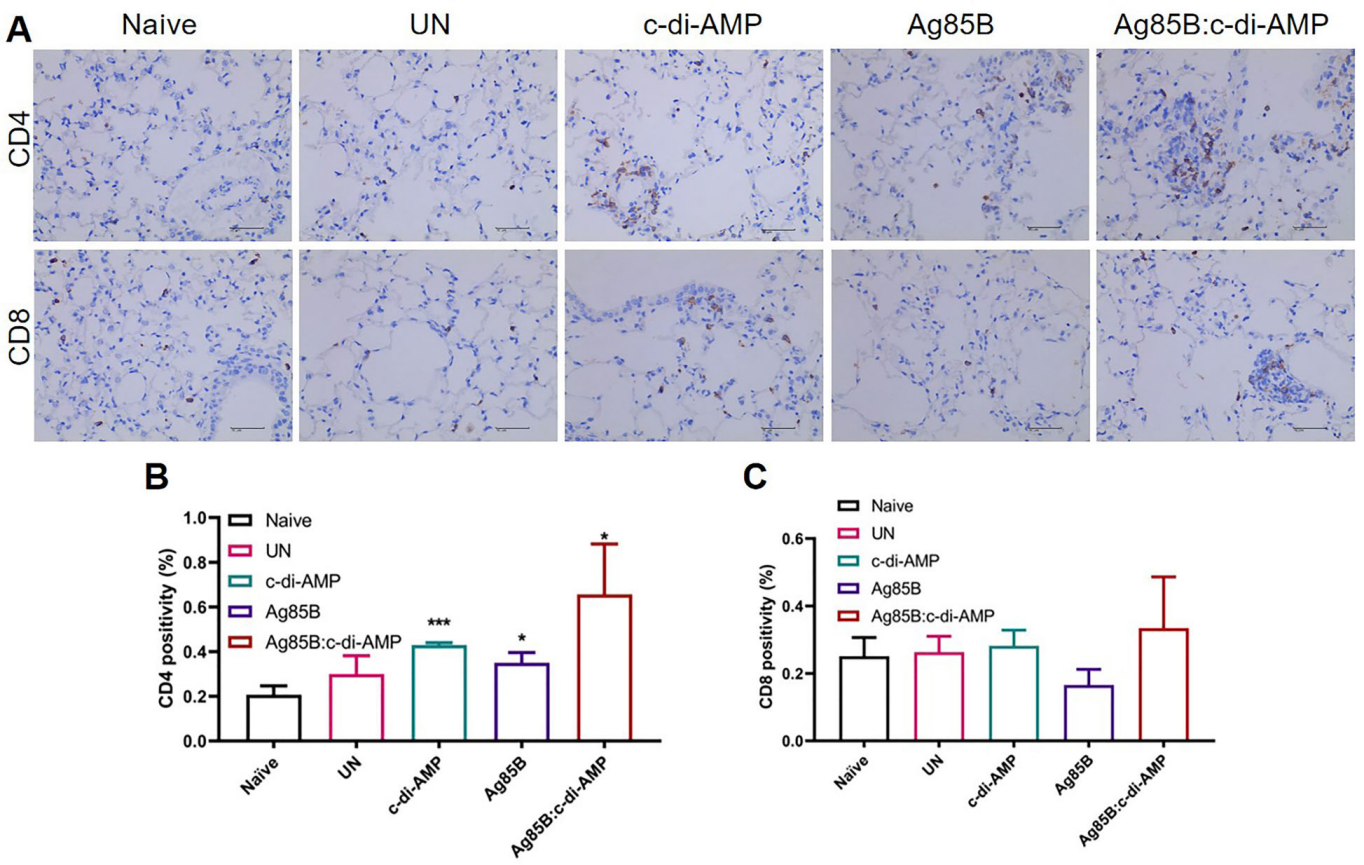
T cell responses in lung of *M. tuberculosis*-infected mice. **A**, CD4^+^ and CD8^+^ T cells distribution in lung tissue sections were determined using immunohistochemistry (400×, scale bar=100 µm) (n=3). Quantitative analysis of CD4^+^ (**B**) and CD8^+^ (**C**) T cell percentages was done using ImageJ software (n=3). Data are reported as means±SE. *P<0.05, ***P<0.001 compared to Naive; Student's *t*-test. UN: infected unimmunized.

CD4^+^ T cells play a crucial role during primary *M. tuberculosis* infection, and TB vaccines increase CD4^+^ T cell responses (24). At 52 weeks, *M. tuberculosis* infection caused a tendency for higher CD4^+^ T cells in mice lung (Figure 4A and B). Ag85B and c-di-AMP alone or combined all stimulated CD4^+^ T cell recruitment in the lung, especially the c-di-AMP alone group (P<0.001). No variations in CD8^+^ T cells were observed among groups (Figure 4A and C).

### Ag85B:c-di-AMP immunization enhanced Th1/Th2/Th17 profile responses in lung

**Figure 5:**
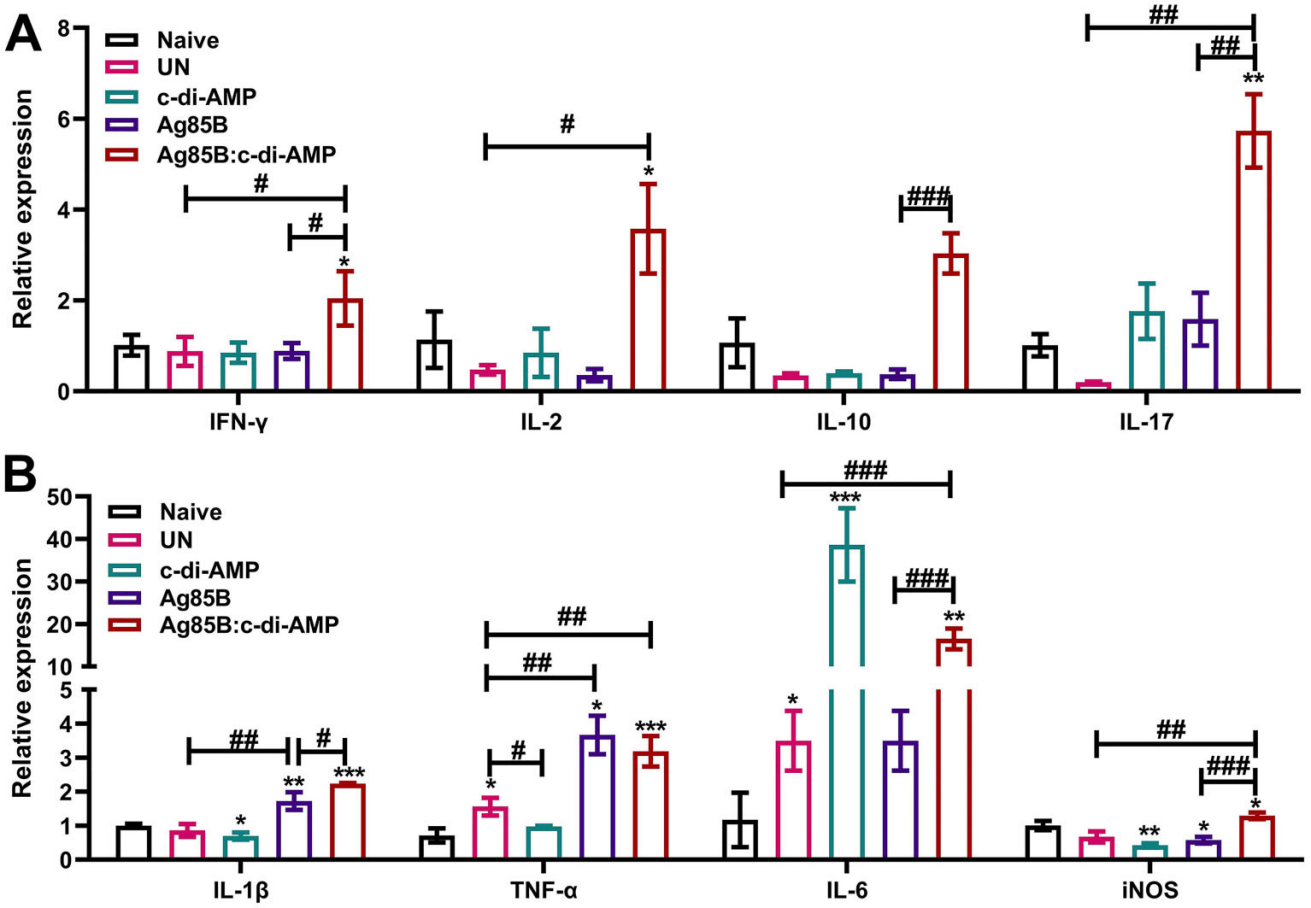
Th1/Th2/Th17 profile responses and inflammatory responses in lung of *M. tuberculosis*-infected mice. The mRNA levels of interferon (IFN)-γ, interleukin (IL)-2, IL-10, IL-17 (n=3) (**A**) and IL-1β, tumor necrosis factor (TNF)-α, IL-6, iNOS (**B**) in the lungs were examined using qRT-PCR (n=3). Data are reported as means±SE. *P<0.05, **P<0.01, ***P<0.001 compared to Naive; ^#^P<0.05, ^##^P<0.01, ^###^P<0.001 as indicated; Student's *t*-test. UN: infected unimmunized.

Ag85B:c-di-AMP stimulated stronger IFN-γ, IL-2, IL-10, and IL-17 expressions in the lung than other groups, which was due primarily to c-di-AMP, as Ag85B alone did not increase Th1/Th2/Th17 profile cytokines expressions in the lung (Figure 5A).

### Ag85B:c-di-AMP immunization enhanced inflammatory cytokines responses in the lung

As *M. tuberculosis* infection causes chronic inflammation, infected mice showed increased TNF-α and IL-6 levels in the lung after 52 weeks of infection. Ag85B alone up-regulated the transcription of IL-1β (P<0.01) and TNF-α (P<0.05) compared to the UN group and had little effect on IL-6 expression in the lung of *M. tuberculosis*-infected mice. Ag85B:c-di-AMP immunization enhanced inflammatory responses of IL-1β (P<0.05), IL-6 (P<0.001), and iNOS (P<0.001) induced by *M. tuberculosis* infection compared with Ag85B alone (Figure 5B). These data demonstrated that Ag85B immunization enhanced inflammatory cytokine responses in persistent mouse *M. tuberculosis* infection. Moreover, c-di-AMP as the mucosal adjuvant significantly increased expression of diverse inflammatory cytokines in *M. tuberculosis-*infected mice.

### Ag85B:c-di-AMP immunization alleviated the pathological lung lesion induced by *M. tuberculosis* infection

**Figure 6:**
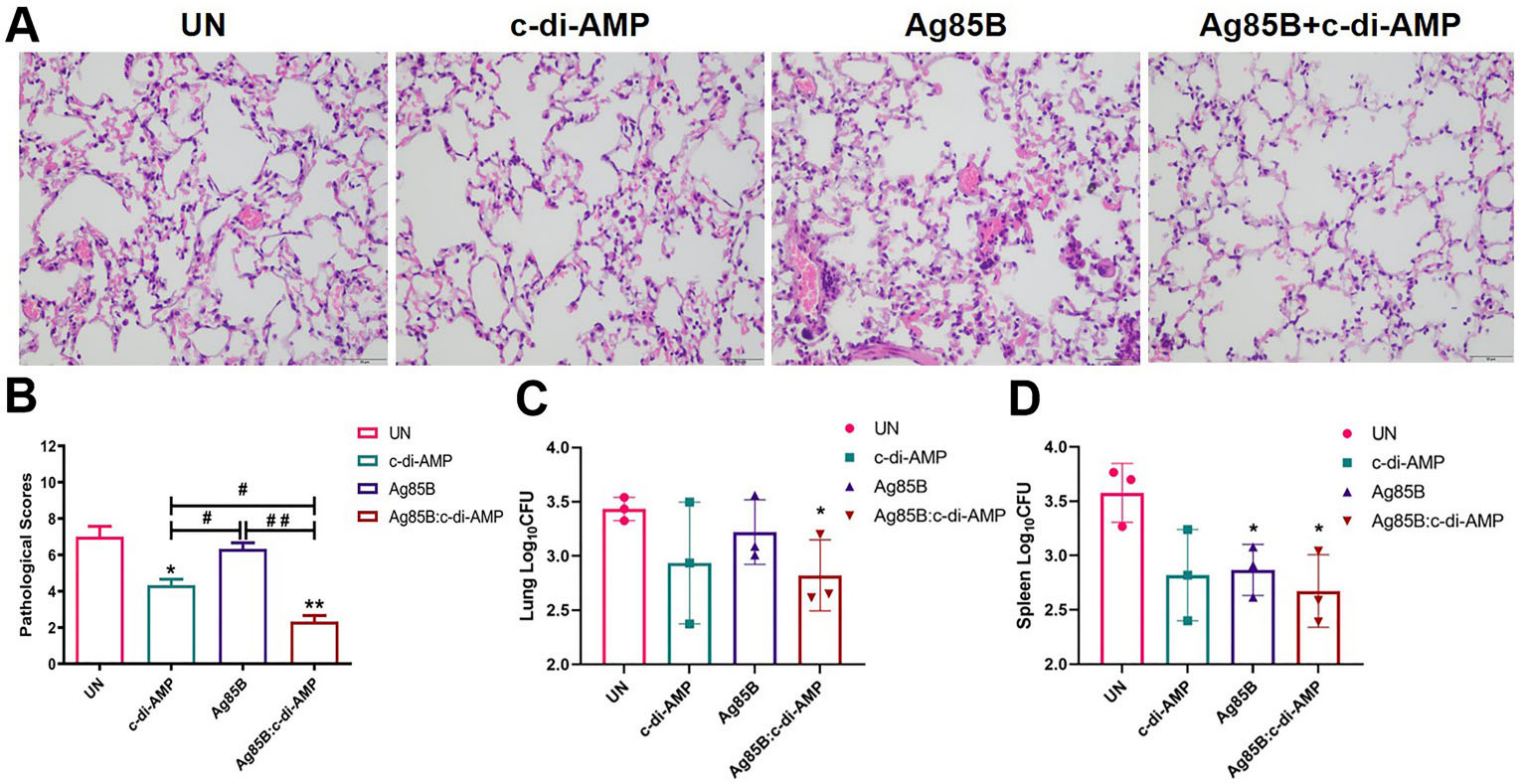
Pathological changes and bacterial burden of *M. tuberculosis*-infected mice. **A**, Pathological section analysis of lung by HE staining (10×40 magnification, scale bar=50 µm) and **B**, pathological scores of lungs at 52 weeks (n=3). Colony forming units (CFUs) in the lungs (**C**) and spleen (**D**) were determined by plate count and are shown as Log_10_ CFU (n=3). Data are reported as means±SE. *P<0.05, **P<0.01 compared to UN; ^#^P<0.05, ^##^P<0.01 as indicated; Student's *t*-test. UN: infected unimmunized.

It is reported that excessive inflammatory responses may result in immunopathological damage of tissues, which may harm the host's immunity and change the outcome of infection (25). *M. tuberculosis*-infected mice showed chronic inflammatory pathological changes, including thickened alveolar septum, patch-like bleeding in alveolar space, disappearance of tracheal cilia, and inflammatory cell infiltration in the UN group by the HE staining. Ag85B immunization relieved the inflammatory responses induced by *M. tuberculosis* infection, but caused more bleeding between alveoli. c-di-AMP vaccination caused infiltration of inflammatory cells and partial alveolar collapse, while Ag85B:c-di-AMP reduced bleeding and inflammatory cell infiltration in *M. tuberculosis*-infected mice (Figure 6A). Ag85B, c-di-AMP, and Ag85B:c-di-AMP immunization all reduced inflammatory pathological changes of *M. tuberculosis*-infected mice, and the effect of Ag85B:c-di-AMP combined the beneficial effects of Ag85B and c-di-AMP, as reflected by pathological scores in lung pathology biopsy (Figure 6B).

### Ag85B:c-di-AMP immunization reduced bacterial loads in *M. tuberculosis-*infected mice

In this study, mice were infected with 5×10^4^ CFUs of *M. tuberculosis* H37Ra by *iv* (14). After 52 weeks of infection, bacterial loads in spleen and lung were about 3.5 log_10_ CFU without TB symptoms, which caused weight loss and appetite loss. Intranasal immunization with either c-di-AMP or Ag85B reduced bacterial loads in the lungs, but not significantly. In lungs, the Ag85B:c-di-AMP group showed 0.40 log_10_ CFU, which was less than that of the Ag85B group, and a significant reduction of bacterial CFUs (P<0.05) than UN mice (Figure 6C). In spleen, c-di-AMP immunization led to 0.71 log_10_ CFUs reduction compared with UN mice, but it was not significantly different between the two groups. Ag85B and Ag85B:c-di-AMP immunization significantly reduced bacterial loads in mice compared with UN mice (P<0.05) (Figure 6D). Overall, Ag85B:c-di-AMP-immunized mice showed the lowest CFUs in lung and spleen among all the *M. tuberculosis*-infected groups. Taken together, intranasal administration of Ag85B:c-di-AMP conferred immunotherapy effects in persistent *M. tuberculosis* infection in mice.

## Discussion

Besides the potential side effects of drugs and the associated financial burden, long-term use of anti-TB drugs has been shown to expedite the development of drug resistance, induce lung inflammation, and promote lung diseases (26). Host immunity is the most important factor in inhibiting the growth of *M. tuberculosis* and LTBI relapse to active *M. tuberculosis* infection (27). The purpose of therapeutic vaccination is the adjunctive treatment of TB or the prevention of LTBI relapse (28). Several therapeutic vaccine candidates, including subunit vaccines with Ag85B as a component, have been approved for testing in clinical trials. In this study, we showed that Ag85B and Ag85B:c-di-AMP induced enhanced immune responses and provided immunotherapeutic effects in a mouse model of persistent *M. tuberculosis* H37Ra infection.

Ag85B is one of the immunodominant antigens of *M. tuberculosis* and the most studied antigen in vaccine candidates. It was reported that Ag85B expression was reduced in persistent *M. tuberculosis* infection, and Ag85B-induced immunity leads to a non-proliferative disease state, which is considered to be the main cause of LTBI (29). Humoral immunity mediated by B cells is generally considered to be protective against extracellular pathogens. Recently, increasing evidence has shown that antibodies have protective effects in intracellular pathogen infection (30). Lower antibody levels were detected by serological studies in both children and adults with extrapulmonary active or disseminated TB (31). As shown in this study, anti-Ag85B antibodies were elevated in serum of *M. tuberculosis-*infected mice, but the level was very low, as that of in TB patients. Ag85B and Ag85B+c-di-AMP vaccination increased antibody levels compared to *M. tuberculosis*-infected mice. IgG subclasses have variable roles in facilitating interaction with other immune cells during *M. tuberculosis* infection. IgG1 plays a predominant protective role in anti-TB humoral responses, which could stimulate the production of TNF-α by monocytes in TB patients (32). A study reported that the candidate vaccine AEC/BC02 (Ag85b+ESAT6-CFP10+CpG with aluminum salt adjuvant) could induce high titers of antigen-specific IgG, IgG1, and IgG2a antibodies, which contributed to its therapeutic effect in this drug-treated LTBI mice model (33). In this study, Ag85B immunization enhanced IgG1 levels in *M. tuberculosis*-infected mice, either alone or with c-di-AMP as an adjuvant.

Cellular immune responses are thought to be the most important defense of humans and animals after *M. tuberculosis* infection (34). Th1, Th2, and Th17 cells are involved in protection against *M. tuberculosis* infection. Th1 cells activate effector functions in macrophages that control intracellular *M. tuberculosis*, and Th17 cells have the capacity to recruit monocytes and Th1 lymphocytes to the site of granuloma formation against TB at early stages (35). As a mucosal adjuvant, c-di-AMP enhanced Th1, Th2, and Th17 immune responses induced by antigens in normal mice (14). In lung, Ag85B:c-di-AMP caused aggregation of CD4^+^ T cells, and significantly enhanced Th1/Th2/Th17 responses compared to Ag85B alone, which confirmed that c-di-AMP as a mucosal adjuvant could enhance antigen-specific immune responses (14). In this study, *M. tuberculosis* infection in mice induced higher CD4^+^ cell levels in the lung and lower CD8^+^ T cell levels in the spleen, indicating a disparity of cellular immunity in different tissues. This could be the result of mucosal immunization, as it has been reported that H4 and H28 mycobacterium antigen fusion was mainly concentrated in the lungs and provided protection against aerosol *M. tuberculosis* infection in mice (36).

The greatest concern of immunotherapy vaccines is their safety and efficacy for clinical use. Ag85B alone caused bleeding in the lungs, detected by HE staining, while Ag85B:c-di-AMP inoculation appeared mildly inflammatory, suggesting that the inflammatory level did not exacerbate pathological damage. We speculated that the lower immunopathological damage may be related to the discontinuous, self-limited regulation mechanism of c-di-AMP (37). Overall, the weight of mice in each vaccination group was comparable to that of normal control mice, indicating that these vaccines did not cause significant side effects such as weight loss. Thus, Ag85B is a safe antigen for immunotherapy vaccines. c-di-AMP alone had a certain therapeutic effect in mice, which may be attributed to its capacity to stimulate the innate and adaptive immunity, as previously reported (38). c-di-AMP as a potential mucosal adjuvant may also be used in therapeutic or prophylactic vaccine strategies for other respiratory infectious diseases.

Previously, we reported that a recombinant *M. smegmatis* expressing Ag85B-ESAT6 showed immunotherapeutic effects in a modified Cornell mouse model of LTBI induced with H37Rv strain by subcutaneous inoculation (39). However, it was difficult to evaluate TB vaccines in a biosafety level (BSL)-3 laboratory after the outbreak of COVID-19, so a persistent infection mouse model with *M. tuberculosis* H37Ra was used in BSL-2 conditions, as previously reported (40). We found that there was no significant difference in immune response levels and bacterial loads between H37Rv and H37Ra for 2 to 10 months (Ning H, Kang J, Lu Y, Bai Y, unpublished results). In this study, we set up a 32-week *M. tuberculosis* H37Ra infected-mouse model, which maintained low bacterial load and weight, and was asymptomatic until the end of experiments at 52 weeks, similar to a LTBI phenotype. Due to many reasons such as experimental design, animal feeding costs, and experimental conditions, this study kept the number of animals at a statistical minimum. Based on the results of this study, we will further explore the immunotherapy effect of Ag85B:c-di-AMP in H37Rv infection model with more animals under different strategies, as soon as the BSL-3 laboratory is available.

In summary, administration of two Ag85B vaccines induced anti-*M. tuberculosis* immune responses for 20 weeks, and Ag85B:c-di-AMP immunization induced higher immune responses and significantly reduced bacterial loads in spleen and lungs, showing that Ag85B:c-di-AMP may be beneficial as an adjunct therapy for TB therapy or as a preventive agent in the recurrence of persistent *M. tuberculosis* in TB patients and LTBI.

## Supplementary Material

Click here to view [pdf].
