# Supplementary material for: Ag85B with c-di-AMP as mucosal adjuvant showed immunotherapeutic effects on persistent Mycobacterium tuberculosis infection in mice
Source: Braz J Med Biol Res. 2024 Jul 1;57:e13409. doi: 10.1590/1414-431X2024e13409 (PMC11221865; doi:10.1590/1414-431X2024e13409)

Figure S1. Purification of Ag85B protein. **A**, Expression and purification of Ag85B analyzed by SDS-PAGE. Un-induced (lane 1), IPTG-induced (lane 2) recombinant *E. coli* DH5α lysates, and Ag85B elutions (lanes 3 to 5). **B**, Analysis of Ag85B after dialysis by SDS-PAGE.

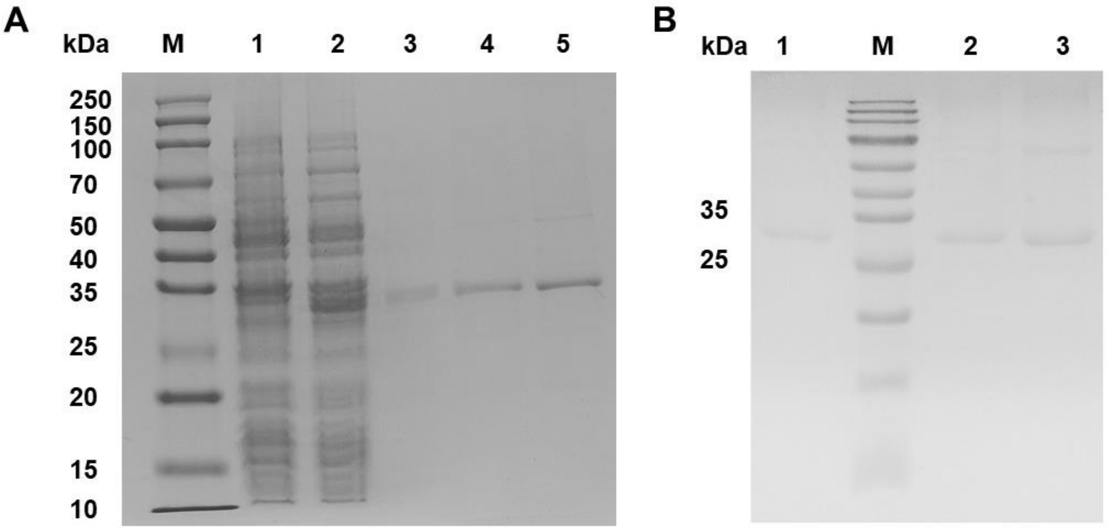

Figure S2. Weight changes of mice after *M. tuberculosis* infection. Mice were infected with *M. tuberculosis* (Mtb) by the intravenous route at week 0 (n=12), and normal naive mice were used as control (n=3).

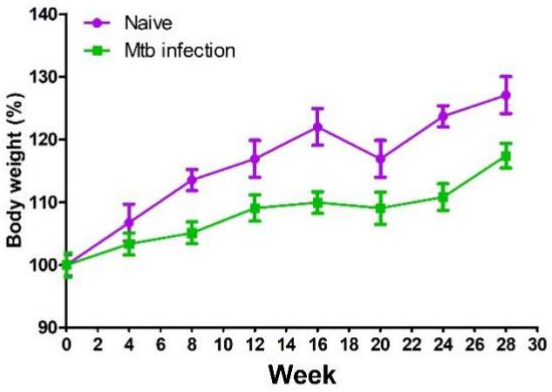

Supplement: Supplementary file 1 [file 1414-431X-bjmbr-57-e13409-suppl.pdf]
